# SG-APSIC1149: Continuous quality improvement project: Changing from sterile to clean perineal care sets at Maharaj Nakorn Chiang Mai Hospital

**DOI:** 10.1017/ash.2023.99

**Published:** 2023-03-16

**Authors:** Prapaipan Wongkreua, Narumon Lerskornsan

**Affiliations:** Maharaj Nakorn Chiang Mai Hospital, Chiang Mai, Thailand; Maharaj Nakorn Chiang Mai Hospital, Chiang Mai, Thailand

## Abstract

**Objectives:** The central sterile supply department (CSSD) provides sterile perineal care sets (SPC sets) for use with patients. However, the SPC sets exceed the standard, and the sterilization process incurs high cost. Therefore, a CSSD nursing team set out to find ways to save costs by providing clean perineal care sets (CPC sets) instead of sterile sets. We examined the rate of catheter-associated urinary tract infection (CAUTI) after using CPC sets, measured the satisfaction of the nursing staff who use the CPC sets, evaluated the decrease in the cost to the hospital. **Methods:** The CSSD nursing team presented some evidence of the benefits of using the CPC sets to the infection control subcommittee and asked for their approval to use CPC sets instead of SPC sets. After approval by the subcommittee, the CSSD nursing staff began to use CPC sets for patients. The incidence of CAUTI was monitored, and a satisfaction survey of the nurses who used the CPC sets was performed. We compared the costs between the SPC set and the CPC set to determine the cost savings. **Results:** The CAUTI rate did not change after using CPC sets. The nurses who used the CPC sets indicated no difference in satisfaction between the SPC and CPC sets, and the cost of the CPC set was cheaper than the SPC set (27 Baht per set). **Conclusions:** In a quality improvement effort, using the CPC set was safe for patients. The users were satisfied with the CPC set and trusted the safety of the instruments. Moreover, using the less expensive CPC sets saved the hospital >700,000 Baht per year.

